# Mechanisms for Curing Yeast Prions

**DOI:** 10.3390/ijms21186536

**Published:** 2020-09-07

**Authors:** Lois E. Greene, Farrin Saba, Rebecca E. Silberman, Xiaohong Zhao

**Affiliations:** 1 Laboratory of Cell Biology, National Heart Lung Blood Institute National Institutes of Health, Bethesda, MD 20892, USA; farrin.saba@nih.gov (F.S.); res@mit.edu (R.E.S.); zhaox@nhlbi.nih.gov (X.Z.); 2 Koch Institute for Integrative Cancer Research, Depart Biol., Howard Hughes Medical Institute, MIT Cambridge, MA 02139, USA

**Keywords:** prion, yeast, mechanism, curing, Hsp104, methods

## Abstract

Prions are infectious proteins that self-propagate by changing from their normal folded conformation to a misfolded conformation. The misfolded conformation, which is typically rich in β-sheet, serves as a template to convert the prion protein into its misfolded conformation. In yeast, the misfolded prion proteins are assembled into amyloid fibers or seeds, which are constantly severed and transmitted to daughter cells. To cure prions in yeast, it is necessary to eliminate all the prion seeds. Multiple mechanisms of curing have been found including inhibiting severing of the prion seeds, gradual dissolution of the prion seeds, asymmetric segregation of the prion seeds between mother and daughter cells during cell division, and degradation of the prion seeds. These mechanisms, achieved by using different protein quality control machinery, are not mutually exclusive; depending on conditions, multiple mechanisms may work simultaneously to achieve curing. This review discusses the various methods that have been used to differentiate between these mechanisms of curing.

## 1. Introduction

Yeast has long served as a model system for studying neurodegenerative diseases, including prion disease, due to cellular pathways being highly conserved from yeast to mammals plus yeast having the advantages of a short generation time and ease of genetic manipulation. Prion proteins exist in two conformations, a normal folded conformation and a misfolded prion conformation, the latter of which is typically characterized by the occurrence of β-rich amyloid fibrils. It is the misfolded conformation that is the infectious agent in prion disease [1]. In mammalian cells, the prion protein is mostly localized on the cell surface, tethered to the plasma membrane through a C-terminal glycosylphosphatidylinositol (GPI) membrane anchor [2,3]. The mammalian prion protein traffics along the endolysosomal pathway either to be recycled to the plasma membrane or degraded in the lysosome. This trafficking pathway enables the misfolded prion protein to spread via exosomes and cell–cell contact [4,5]. Due to the complexities of studying prion protein in mammalian cells, the study of yeast prions has been particularly useful in furthering our understanding of the growing number of prion-related diseases. In yeast, the misfolded prion proteins exist as amyloid fibrils or prion seeds, which are transmitted during mating and cell division [6]. To maintain a steady-state number of prion seeds from generation to generation, there must be constant propagation of the prion seeds, which is most commonly dependent on the severing activity of the molecular chaperone Hsp104. Hsp104 is a ring-forming disaggregase, composed of six Hsp104 protomers, with each protomer containing two triple-A nucleotide binding domains.

The most widely studied prions in *Saccharomyces cerevisiae* are [*PSI*^+^], [*URE3*], and [*PIN*^+^], which are the misfolded protein conformations of Sup35, Ure2, and Rnq1, respectively [7]. The properly folded Sup35 and Ure2 proteins have a well-defined function in yeast, but the function of Rnq1 is not known. Sup35 is an essential protein that is part of the translation termination complex, while Ure2 represses transcription of genes involved in nitrogen catabolism. The misfolded prion proteins have been shown to exist as variants [8] that differ in their structure and biophysical properties [8,9,10,11]. The weaker variants have fewer seeds, greater thermostability, and are severed more poorly than the stronger variants. When the prion seeds are completely eliminated, the yeast are cured, and since the weak variants have fewer seeds, they are cured more readily than the strong variants [11]. Several different mechanisms have been found to cure yeast prions. These mechanisms, which are not mutually exclusive, include inhibition of severing, dissolution of the prion seeds, asymmetric segregation of the prion seeds, and proteolysis of the prion seeds.

Before discussing the various methods for curing yeast prions, it should be noted that our understanding of curing is dependent on our ability to distinguish between the normal folded conformation and the amyloid conformation of prion proteins. In [*PSI*^+^], the two conformations can be distinguished in yeast with a nonsense mutation in the adenine biosynthetic genes. When Sup35 is in its amyloid conformation, there is enough read-through of stop codons to prevent accumulation of intermediates in the adenine pathway, resulting in colonies having a white color. However, when Sup35 is in the normal folded conformation, the nonsense mutations cause intermediates in the adenine synthesis pathway to accumulate, which causes the colony to have a red color [12]. This assay has been modified to be used as a read out for both the [*URE3*] and the [*PIN*^+^] prions [13,14]. In the former case, the *ADE2* gene, part of the adenine synthesis pathway, is controlled by the *DAL5* regulatory region, which is strongly repressed by Ure2. In the latter case, the prion domain of Rnq1 was fused to the M- and C-domains of Sup35. Another method to distinguish the amyloid prion conformation from the normal folded protein in yeast lysates is a sedimentation assay that separates the high molecular weight amyloid in the pellet from the soluble normal form in the supernatant, followed by sodium dodecyl sulfate (SDS) gel electrophoresis of the fractions [15]. Alternatively, running partially denatured lysates on semi denaturing detergent (SDD) agarose gels has been used to separate by mobility the high molecular weight aggregate from the properly folded protein [16]. Finally, fluorescence labeling of the prion protein has also been proven very useful to distinguish the amyloid conformation by fluorescence microscopy, which appears as distinct foci in the cytosol, from its normal folded conformation, which appears diffuse [17].

## 2. Curing by Inactivation of Severing of the Prion Seeds

**Figure 1:**
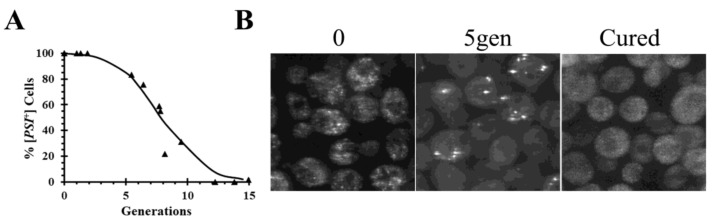
Curing of [*PSI*^+^] in SY80 yeast strain by overexpression of dominant negative Hsp104 mutant. (**A**) Time course of curing [*PSI*^+^] as a function of generation time. (**B**) Images of the green fluorescent labeled (GFP)-labeled foci in SY80 yeast strain before induction, after 5 generations in galactose medium to induce expression of the dominant negative mutant, and completely cured. SY80 strain contains a relatively strong [*PSI*^+^] variant, which was engineered to express GFP-labeled Sup35 from the *SUP35* chromosomal locus [26].

The most widely used and best understood method for curing the canonical prions, which are characterized by having a misfolded amyloid structure, is by inactivation of severing. The seeds are normally severed by Hsp104 [18], although in a few cases, severing can be carried out by other proteins. In addition to Hsp104, severing of the prion seeds is dependent on Sis1, a member of the Hsp40 family that binds to Hsp70 through its J-domain, and Ssa1, a member of the Hsp70 family. The binding of the Sis1–Ssa1complex activates Hsp104 [19,20,21], so impairing the activity of Sis1, Ssa1, or Hsp104 can cure the prions. Inactivation of Hsp104 activity is the most common method to cure prions, which can be accomplished by addition of millimolar concentrations of guanidine or by overexpressing a dominant negative mutant, which inhibits the function of the wild-type protein. Low concentrations of guanidine inactivate by binding to aspartic acid (D184) of Hsp104 [22], while mutating the Walker motifs in each nucleotide binding domain inactivates the ATPase activity of Hsp104 [18,23]. Since these two methods inactivate the severing of the prion seeds by Hsp104, they yield similar kinetics of prion curing. In addition, prion curing has also been achieved by conditionally deleting *HSP104* since Hsp104 is a non-essential gene [24]. When Hsp104 is inactivated, there is a lag before the onset of curing, as shown by the curing curve in Figure 1A in which the data are plotted as percent yeast cells with prion versus generation time. During this lag phase, the seeds are halved each generation as the yeast divide. Due to the volume ratio between the mother and daughter cells, about 60% of the prion seeds remain in the mother cell while 40% of the seeds are transmitted to the daughter cell at cell division [25]. The length of lag phase is determined by the number of seeds initially present in the cells and are only cured when yeast are completely devoid of seeds.

In addition to following the curing curves, fluorescence imaging of the prion proteins labeled with green fluorescent protein (GFP) has also been used to monitor the curing of the prions by Hsp104 inactivation in real-time. When a [*PSI*^+^] yeast strain was cured by overexpression of a dominant negative Hsp104 mutant, there is a reduction in the number of foci as a function of generation time (Figure 1B) consistent with the lack of severing [24]. During the curing, the foci become brighter, perhaps due to binding of the newly synthesized Sup35 molecules to the ends of the filaments. With continued inactivation of Hsp104 activity, all the foci are eliminated, at which time the fluorescence becomes diffuse and the yeast are cured.

As discussed above, inhibiting the severing of the prion seeds has also been achieved by interfering with either Sis1 or Ssa1 activity. Since Sis1 is an essential protein, curing of different prions has been achieved by reducing the Sis1 concentration in the cell using a Tet-off inducible promoter [27]. Although this reduction in Sis1 concentration cures the canonical prions, the time course is quite different from that of Hsp104 inactivation since the Sis1 is not completely depleted. For example, it took ~60 generations for partial depletion of Sis 1 to cure the [*PSI*^+^] prion, while inactivation of Hsp104 with guanidine cured [*PSI*^+^] in ~10 generations [27,28]. There is also curing of [*URE3*] and some variants of [*PIN*^+^] by depleting Ssa1, which has been achieved by overexpressing Ydj1, a member of the Hsp40 family [29,30,31]. Ssa1 is depleted by binding to the J-domain of Ydj1, as shown by the lack of [*URE3*] curing when Ydj1 is mutated at its J-domain to prevent its binding to Ssa1 [29]. However, the extent of Ssa1 depletion is evidently not sufficient to completely inhibit Hsp104 activity since overexpression of YDJ1 does not cure [*PSI*^+^] [32].

## 3. Curing by Dissolution

**Figure 2:**
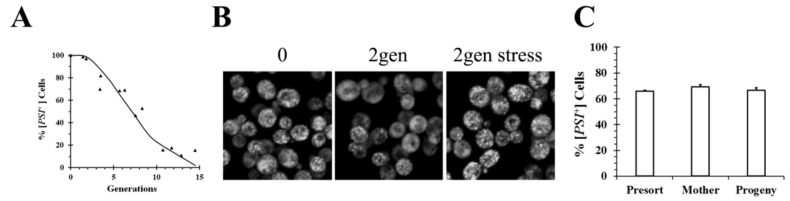
Curing of [*PSI*^+^] in SY80 yeast strain by overexpression of wild-type Hsp104. (**A**) Time course of curing [*PSI*^+^] as a function of generation time. (**B**) Images of the GFP-labeled foci in SY80 before induction, after 2 generations in galactose medium to induce Hsp104 expression, and 2 generations in galactose medium, followed by stressing the cells by starvation. (**C**) Flow cytometry analysis of curing of [*PSI*^+^] in 779-6a strain.

Another mechanism of prion curing is achieved by gradually decreasing the size of the prion seeds until they completely disassemble. One way this could occur is for the monomers that make up the prion seeds to gradually dissociate from the ends of the seeds or amyloid fibers. Such a mechanism has been proposed to explain the paradoxical observation that overexpression as well as inactivation of Hsp104 cures [*PSI*^+^], an observation made more than two decades ago by Chernoff et al. [18]. To investigate the mechanism of [*PSI*^+^] curing by Hsp104 overexpression, imaging was used to examine the fluorescent seeds in different [*PSI*^+^] strains. As shown in Figure 2, early after addition of galactose to induce Hsp104 overexpression, the GFP fluorescence of Sup35 appeared quite heterogenous. In some cells, the seeds or foci are clearly visible, whereas in other cells, they are no they are no longer resolvable by microscopy although the cells are all [*PSI*^+^] based on the plating assay. Since the foci are evident in some cells and not in other cells, as well as being visible in all cells at the start of the experiment, rapid movement of foci during the curing process does not appear to explain the lack of foci in some of the cells. The prion seeds become visible again after the cells are stressed by starvation, which perhaps causes an increase in the size of the prion seeds by altering the chaperone balance in the cell. Therefore, the seeds are present all along but were too small to resolve unless their size was increased by stressing the cells (Figure 2B).

These results for the curing by Hsp104 overexpression are consistent with a similar observation obtained when we examined the curing of [*PSI*^+^] by guanidine inactivation of the Hsp104 severing activity. In contrast to overexpressing the dominant negative Hsp104 mutant, early in the curing process when the severing activity of Hsp104 was inhibited by addition of millimolar concentrations of guanidine, the foci could not be resolved by microscopy in cells even though the plating assay showed the cells were all [*PSI*^+^]. Stressing the cells made the foci visible, just as was observed with curing by Hsp104 overexpression. Therefore, the absence of resolvable foci during curing suggests that in addition to severing activity, which is inhibited by guanidine, Hsp104 has another activity, which we called trimming, which is not inhibited by guanidine. In contrast to severing activity, which reduces the number of seeds, the trimming activity does not affect the number of seeds. Instead, trimming activity gradually reduces the size of the seeds, an effect that is reversed by stressing the cells. The ability of Hsp104 to trim the prion seeds suggests that this as a mechanism for curing [*PSI*^+^] by Hsp104 overexpression. Curing of [*PSI*^+^] occurs by Hsp104 gradually trimming the prion seeds until they are completely disassembled. Dissolution of the prion seeds as a mechanism for curing [*PSI*^+^] by Hsp104 overexpression has been previously proposed by Paushkin et al. [15] based on sedimentation assays.

Further evidence that the curing of [*PSI*^+^] by Hsp104 overexpression is due to the trimming of the prion seeds comes from finding mutants of Hsp104 that have lost their trimming activity, which was determined by imaging the [*PSI*^+^] foci in the presence of millimolar concentrations of guanidine, and showing that overexpression of these Hsp104 mutants no longer cures [*PSI*^+^]. Two different point mutants of Hsp104 were found not to trim the prion seeds in guanidine, Hsp104(T160M) and Hsp104 (D184S). In agreement with previous results [33], overexpression of Hsp104(T160M) was found not to cure [*PSI*^+^], and also not to trim the prion seeds [34]. Similarly, overexpression of the Hsp104(D184S) mutant did not cure [*PSI*^+^] or trim the prion seeds [34]. A role for trimming in curing of [*PSI*^+^] by Hsp104 overexpression was also obtained when we overexpressed Hsp104 homologs from either *Candida albicans* or *Schizosaccharomyces pombe*. These homologs neither trimmed the prion seeds nor cured the strong [*PSI*^+^] variants, but they did trim the prion seeds and cure the weak [*PSI*^+^] variants [35]. Here, again, there is no curing of [*PSI*^+^] by Hsp104 overexpression in the absence of trimming.

Another mechanism in which Hsp104 overexpression could lead to dissolution is by increasing fragmentation of the [*PSI*^+^] seeds, which would ultimately cause their dissolution. This proposal is supported from in vitro studies that showed that increasing Hsp104 concentration caused fragmentation of amyloid fibers, composed of the N-terminal prion domain, the domain that forms the amyloid core, and the M-domain of Sup35 [36]. Since this mechanism depends on severing of the prion seeds by Hsp104, then inhibiting the severing activity of Hsp104 with millimolar concentrations of guanidine should decrease the rate of curing [*PSI*^+^] by Hsp104 overexpression. However, since guanidine did not significantly affect the rate of curing of [*PSI*^+^] by Hsp104 overexpression [34,37], it is difficult to reconcile the in vivo data with a fragmentation mechanism for curing.

Aside from trimming causing a reduction in seed size, a size reduction would also occur if monomer addition to the seeds was inhibited, but the seeds were still being severed. In this case, the seeds would continuously decrease in size until they completely disassembled, and the yeast were cured. This mechanism of curing may account for how overexpression of human DnaJB6, a member of the Hsp40 family, cures both [*URE3*] and weak [*PSI*^+^] variants [38,39]. Imaging studies with both [*URE3*] and weak [*PSI*^+^] variants showed that DnaJB6 overexpression caused a loss of resolvable foci or seeds in cells that were still not cured, suggesting that in both cases, the seeds were becoming smaller before they completely disappeared [39]. The observation that DnaJB6 inhibits nucleation of the Ure2 protein in biochemical assays using purified proteins is consistent with DnaJB6 blocking the amyloid seeds from growing larger, but not affecting their severing [38]. Overexpression of the two small heat shock proteins, Hsp26 and Hsp42, has also been proposed to cure [*PSI*^+^] by reducing the size of the prion seeds [40]. Like DnaJB6, Hsp26 is proposed to block the growth of amyloid fibers, whereas Hsp42 has been proposed to inhibit the conformational rearrangements that generate the amyloidogenic oligomers, in effect blocking the growth of the amyloid fibers.

Finally, the Manogaran laboratory recently reported that the chemical reagent dimethyl sulfoxide (DMSO) cures the weak [*PSI*^+^] variants by protein solubilization involving an increase in Hsp104 expression [41]. They found that the rate of curing of the weak [*PSI*^+^] variants by DMSO addition was significantly faster than the rate of curing by Hsp104 inactivation. This curing was accompanied by an increase in Hsp104 expression along with an increase in free Sup35. In addition, even when cell division was arrested by the addition of alpha factor, which inhibits cell division, there was significant curing by DMSO. This suggests that curing may be due to disassembly of the prion seeds since curing by disassembly should depend on time of incubation with the disassembly agent rather than cell division. On the other hand, when [*PSI*^+^] is cured either by Hsp104 inactivation or asymmetric segregation, cell division is necessary to reduce seed number. As they noted, curing by DMSO could be due to disassembly of the prion filament or to accumulation of newly synthesized Sup35 monomer that cannot bind to pre-existing filaments or to newly obtained cured cells in the population.

## 4. Curing by Asymmetric Segregation

Curing of prions by asymmetric segregation has the distinguishing feature that the mother and daughter cells cure at different rates. The most common cause of curing by asymmetric segregation is coalescence of the prion seeds, which, in turn, produces aggregates that are too large to pass through the bud neck of the dividing yeast. Asymmetric segregation has also been attributed to a physical barrier that retains the seeds in the mother cells as well as to unequal chaperone distribution between mother and daughter cells. This mechanism of curing has been reported for all the canonical prions.

**Figure 3:**
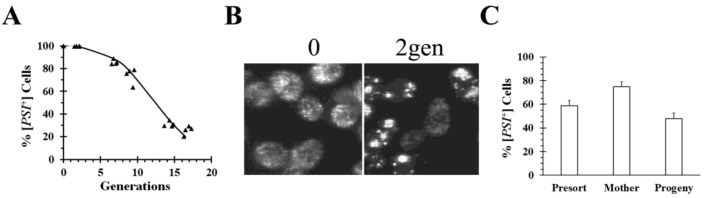
Curing of [*PSI*^+^] in SY80 yeast strain by overexpression of a *S. pombe* Hsp104 fragment, Hsp104ΔC32. (**A**) Time course of curing [*PSI*^+^] as a function of generation time. (**B**) Images of the GFP-labeled Sup35 in SY80 before induction and after inducing *S. pombe* Hsp104ΔC32 expressing for 2 generations with galactose. (**C**) Flow cytometry analysis of curing of [*PSI*^+^] in SY80 strain expressing *S. pombe* Hsp104ΔC32 for 6 generations. Cell wall was fluorescently labeled with wheat germ agglutin, as described previously [34], mother cells were then separated from progeny based on fluorescent labeling by flow cytometry.

There have been numerous examples where asymmetric segregation is due to aggregation of the prion seeds. Overexpression of Btn2 was observed to cure the [*URE3*] prion by asymmetric segregation when the yeast were imaged with either the N-terminal prion domain of Ure2 or full-length Ure2 labeled with GFP [39,42]. Aggregation of the Ure2 seeds has also been observed during the curing of [*URE3*] by overexpression of Hsp42, Cur1, or Ydj1, suggesting that, here too, asymmetric segregation has a role in curing [*URE3*] [39]. Similar to [*URE3*], overexpression of Cur1 aggregates the prion seeds in weak [*PSI*^+^] yeast, which contributes to curing by asymmetric segregation [39]. Aggregation of prion seeds also occurs when Ssa1 is overexpressed in weak [*PSI*^+^] yeast, which, in turn, leads to curing by asymmetric segregation [43]. This was shown by imaging the prion seeds and measuring the size of the oligomers on semi-denaturing gels [43]. However, neither overexpression of Cur1 nor Ssa1 cured the stronger [*PSI*^+^] variants. Another example of asymmetric segregation was observed upon overexpression of a C-terminal Hsp104 truncation mutant from *S. pombe*, Hsp104ΔC32. This fragment cured both the weak and strong [*PSI*^+^] variants by asymmetric segregation (Figure 3), but, surprisingly, a similar phenomenon did not occur with a C-terminal truncation mutant from *S. cerevisiae* Hsp104 [35]. Imaging of cells during curing by overexpression of *S. pombe* Hsp104ΔC32 showed that the [*PSI*^+^] seeds formed large aggregates (Figure 3B). Furthermore, we found that mother cells, which were labeled before expressing Hsp104ΔC32 for 6 generations, cured significantly slower than their progeny when the two populations were isolated by flow cytometry (Figure 3C). This shows that [*PSI*^+^] is being cured by asymmetric segregation. Aggregation of [*PIN*^+^] seeds was also observed by the Manogaran laboratory, who found that this occurred upon disruption of the actin cytoskeleton with a specific [*PIN*^+^] variant [44]. Imaging showed that this causes the appearance of larger [*PIN*^+^] aggregates, suggesting that curing was due to asymmetric segregation of the prion seeds. 

Heat shock has also been found to cure weak [*PSI*^+^] variants by asymmetric segregation [45,46]. Although heat shock caused upregulation of heat shock proteins, including Hsp104, the increase in Hsp104 expression was found not to correlate with the extent of curing [45]. The Chernoff laboratory found that heat shock cured the daughter cells faster than the mother cells, which they proposed was due to greater retention of prion seeds in the mother cells [46]. Although the Serio laboratory also found that heat shock cured weak [*PSI*^+^] by asymmetric segregation, they, surprisingly, observed the opposite asymmetry from the Chernoff laboratory; in their experiments, the mother cells cured faster than the daughter cells. They proposed that there was greater retention of Hsp104 in the mother cells, which, in turn, lead to the mother cells having fewer seeds and faster curing than the daughter cells [45]. The difference between the results from the Chernoff and Serio laboratories has been attributed to growth conditions, i.e., the yeast were in log phase in the experiments from the Serio laboratory, but not in the experiments from the Chernoff laboratory [45].

Finally, contrary to our results showing that Hsp104 overexpression cures [*PSI*^+^] by trimming the prion seeds, the Tuite laboratory suggested that overexpression of full-length Hsp104 cures [*PSI*^+^] by asymmetric segregation [37]. This suggestion was based on micromanipulation to separate budding cells, followed by counting the number of prion seeds in the separated mother and daughter cells [47]. They found that after overexpression of Hsp104 in [*PSI*^+^] cells for 5.7 generations, a time period during which the yeast were ~50% cured, the mother cells had significantly more seeds than the daughter cells, although this unexpectedly was not apparent at 3.6 generations when the yeast were ~30% cured [37]. At the same time, their data indicated that the severing of the prion seeds was not affected by Hsp104 overexpression since the average number of prion seeds per mother and daughter cells together remained constant over the period of HSP104 overexpression. Surprisingly, the mobility of the aggregates on semi-denaturing gels did not change in yeast overexpressing Hsp104 [16]. A decrease in mobility would be expected if there was an increase in the size of the aggregates. Therefore, the observed asymmetric segregation is not due to larger aggregates, which led them to propose that the prion seeds are being retained in the mother cells by anchoring to a sub-cellular structure [37].

In contrast to Tuite’s experiments, data from our laboratory did not detect the occurrence of asymmetric segregation during the curing of [*PSI*^+^] by Hsp104 overexpression. In our experiments, we used flow cytometry to separate different yeast populations, followed by plating to determine the percent of [*PSI*^+^] in each population [34]. No significant difference in curing was found between newborn and old cells in yeast overexpressing Hsp104 for 5 generations after staining bud scars and then using flow cytometry to isolate the two populations. Similarly, we found there was no significant difference in the extent of curing between the mother cells, which were labeled before inducing Hsp104 overexpression for 3 generations, and their progeny when the two populations were isolated by flow cytometry (Figure 2C). Based on their observation of asymmetric segregation, the Tuite laboratory would have predicted that there would be more curing of the newborn cells than the old cells in the first experiment and similarly, there would be more curing of the progeny than the mother cells in the second experiment. More work will be needed to reconcile these differences between our results and those from the Tuite laboratory and thus to understand the mechanism of curing of [*PSI*^+^] by Hsp104 overexpression.

## 5. Curing by Proteolysis

Degradation is an ideal way to clear misfolded proteins, both non-amyloid and amyloid proteins, including prions. Protein degradation in yeast is primarily carried out either by the proteasome or by proteases in the vacuole. Covalent modification of proteins with ubiquitin targets them to the proteasome. The [*LSB*^+^] prion is the misfolded conformation of the Lsb2 protein that in its soluble conformation negatively regulates actin nucleation [48]. The protein levels of Lsb2 are regulated by ubiquitination, which leads to a decrease in its protein level due to proteasomal degradation, and heat shock, which upregulates the cellular Lsb2 concentration [49]. When Lsb2 is upregulated, it forms a metastable amyloid conformation that is attached to cortical actin patches. Mutating the ubiquitination sites on [*LSB*^+^] increases its mitotic stability, indicating that proteasomal degradation of Lsb2 produces curing of this prion [49,50]. The ubiquitin pathway also affects the curing of [*PSI*^+^] by Hsp104 overexpression, although there is no direct evidence for ubiquitination of Sup35 [51,52,53]. Specifically, there is a 1.5-fold increase in the rate of curing of [*PSI*^+^] by Hsp104 overexpression, raising the ubiquitin level in the cell [54]. Conversely, there is a decrease in the rate of curing by Hsp104 overexpression upon deletion of either the ubiquitin-conjugating enzyme Ubc4 [52], or Ump1, a proteasome maturation protein [34]. Although all these data support a role for the proteasome in curing of [*PSI*^+^] by Hsp104 overexpression, its role in curing is not yet understood.

Autophagy is the other major degradation pathway for clearance of misfolded proteins. The autophagosome, a double-membrane vesicle that sequesters cytosol and organelles, delivers proteins to the vacuole for degradation. Although it has not been widely implicated in the clearance of prions, Grant and coworkers have shown that the autophagosome pathway prevents spontaneous formation of both [*PSI*^+^] and [*PIN*^+^] prions [55]. They propose that autophagosomes engulf the misfolded proteins caused by oxidation, including oxidized Sup35, whose structure shifts to favor prion formation. A more direct link of autophagy and prion clearance is shown by recent work from the Manogaran laboratory [41]. In their study of the curing of weak [*PSI*^+^] variants by DMSO, they observed that curing was much slower in yeast with mutants in the autophagy pathway, whereas increasing autophagy did not cure weak [*PSI*^+^] variants. The role of autophagy in the curing of [*PSI*^+^] by DMSO is not yet understood.

## 6. Methods: Pros and Cons

**Figure 4:**
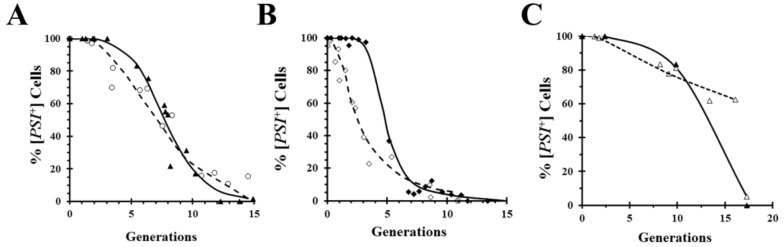
Curing of [*PSI*^+^] by overexpression of dominant negative Hsp104 mutant and wild-type Hsp104 in different strains. Curing by overexpression of dominant negative mutant is shown by solid symbols and solid line, while curing by overexpression of wild-type is shown by open symbols and dashed line. (**A**) Comparison of curing by overexpression of dominant negative mutant (from Figure 1A) and wild-type Hsp104 (from Figure 2A) were obtained using relatively strong [*PSI*^+^] variant in the SY80 yeast strain, a relatively strong variant. (**B**) Comparison of curing by overexpression of dominant negative mutant and wild-type Hsp104 were obtained using the relatively weak [*PSI*^+^] variant in the L2888 yeast strain from the Liebman laboratory [43]. (**C**) Comparison of curing by overexpression of dominant negative mutant and wild-type Hsp104 were obtained using a relatively strong [*PSI*^+^] variant in 779-6a yeast expressing red fluorescent protein (RFP)-labeled Sup35 instead of Sup35 from the *SUP35* chromosomal locus.

A widely used technique for obtaining mechanistic insights into yeast prion curing is to measure the kinetics of curing. Importantly, however, kinetics does not prove that a mechanism of curing is correct, it can only rule out various mechanisms. For example, curing by inactivation of Hsp104 predicts a lag phase while the seeds are being diluted out by cell division, but the presence of a lag phase does not prove that curing is caused by Hsp104 inactivation. This is exemplified by comparing in Figure 4A the curing curves of a relatively strong [*PSI*^+^] variant caused by overexpression of either the dominant negative Hsp104 mutant (Figure 1A) or wild-type Hsp104 (Figure 2A). Although the curing curves are quite similar, the curing is caused by different mechanisms in the two cases. On the other hand, comparison of the curing curves of a weaker [*PSI*^+^] variant in Figure 4B shows a marked difference between the curing curves caused by overexpression of the dominant negative Hsp104 mutant and by overexpression of wild-type Hsp104. As expected, regardless of the [*PSI*^+^] variant, there is a lag phase when [*PSI*^+^] is cured by overexpression of the dominant negative mutant of Hsp104, whereas the curing curves caused by overexpression of wild-type Hsp104 can be quite different depending on the [*PSI*^+^] variant. Specifically, the weak variant cures as soon as Hsp104 overexpression is induced with galactose, unlike the strong variant. This can be attributed to the fact that lower levels of overexpression of wild-type Hsp104 cure weak variants but not strong variants [35]. Thus, after induction of Hsp104 expression, it takes a longer time to reach a sufficiently high Hsp104 level to cure the relatively strong variants.

Curing is typically measured using the red/white colony assay by plating yeast on media containing limiting amounts of adenine, which is needed to keep all cells viable. After depletion of the adenine in the medium, the yeast starts to synthesize adenine, which leads to an accumulation of adenine intermediates and a red color colony when cured yeast are plated, but not in yeast with prions. A limitation of this assay is that there may be curing on the plate, so if this happens, the assay is not a real-time readout of the prion phenotype. This problem occurs when yeast are cured by overexpression of a given protein, since even though plating turns off overexpression of the protein, the protein present must still be diluted out by cell division. Another limitation of the red/white colony assay occurs when curing very weak prion variants, which yield pink rather than white colonies, making it difficult to differentiate cured from non-cured colonies. In the latter case, replica plating colonies onto medium lacking adenine can be used to distinguish cured from non-cured colonies since only yeast with prions will grow in the absence of adenine.

Another assay that has been used to study the mechanism of prion curing is the sedimentation assay, which separates the heavier seeds from the free soluble monomeric protein that does not sediment upon centrifugation of the yeast lysate. SDS gel electrophoresis is then used to quantify the soluble prion protein in the supernatant. However, since it was previously shown that the free monomer protein can bind to seeds in the yeast lysates [56], this could overstate the amount of prion protein in the pellet.

A popular technique to examine the high molecular weight amyloid is to partially denature yeast lysates in sodium dodecyl sulfate at room temperature [16]. The lysates are then run on semi-denaturing detergent agarose gels. This yields high molecular weight bands for the amyloid protein, which do not represent the seeds, but rather the polymers that make up the seeds. Since the conditions are only partially denaturing, the molecular weight of the polymers may be affected by other proteins that bind to the polymers, such as Ssa1/Ssa2 [57]. In addition, both the cell lysis method and electrophoresis conditions can substantially affect the mobility of the polymers on the gel, which, in turn, affects the estimate of the aggregate size [58].

A technique that allows prion curing to be studied in real-time is imaging of fluorescently labeled prion proteins. However, one limitation of imaging is that it requires covalent modification of the prion protein with a fluorescent tag such as GFP, which may affect the properties of the prion. For example, the GFP labeling of Sup35 weakens the [*PSI*^+^] phenotype [35,43]. Surprisingly, when we inserted red fluorescent protein (RFP) between the N-terminal domain and the adjacent M domains of Sup35, the same site that is used for GFP insertion, the [*PSI*^+^] yeast expressing RFP-labeled Sup35 cures by overexpression of the dominant negative Hsp104 mutant, but only shows negligible curing by overexpression of wild-type Hsp104 (Figure 4C). In contrast, overexpression of either the dominant negative mutant or wild-type Hsp104 cures [*PSI*^+^] yeast expressing GFP-labeled Sup35 (Figure 4A,B).

Genetics has been a very powerful tool in yeast cell biology. In the study of prions, it has been used to identify new prions and to find proteins that cure prions. In regard to discovering new prions, Alberti et al. [59] conducted a bioinformatic proteome-wide survey for prionogenic proteins in *S. cerevisiae*. This survey found that out of 100 potential candidates, 19 could form prions and at least one of these 19 prion proteins, Mot3, could misfold into a true prion. As for discovering proteins that cure prions, the Wickner laboratory using a yeast overexpression library found that Btn2 and Cur1 cured [*URE3*] [42]. Interestingly, yeast genetics have shown that even at endogenous expression levels, Btn2 and Cur1 cure many of the de novo formed [*URE3*] prions [60]. The Wickner laboratory also performed another screen using a yeast knockout library that identified two proteins involved in [*PSI*^+^] prion propagation: Siw14, a pyrophosphatase specific for 5-diphosphoinositol pentakisphosphate (5PP-IP5), and the proteins Upf1 and Upf3, components in a complex involved in nonsense-mediated mRNA decay [61,62]. The screen showed that in the deletion strains, more [*PSI*^+^] colonies were formed than in the wild-type strain. As expected, when these proteins were expressed in their respective deletion strain, there was a marked reduction in [*PSI*^+^] colonies, suggesting that these proteins cure some [*PSI*^+^] variants. Similarly, genetics identified a function for the proteasome as well as the cochaperones, Sti1 and Cpr7, in the curing of [*PSI*^+^] by Hsp104 overexpression [54,63].

In vitro studies using purified proteins have provided insights into the mechanism by which chaperones cure prions. Generally, this reductionist approach has used the prion domain or other misfolded substrates such as denatured luciferase, rather than the full-length prion protein. At least when expressed in the cell, the prion domain shows much greater tendency to aggregate than the full-length protein [64,65]. In particular, studies from the Bukau laboratory have greatly enhanced our understanding of the Hsp70 network, which cooperates with numerous other chaperones and participates in both protein aggregation and disaggregation pathways [66]. In addition, biochemical studies of Hsp104 with different nucleotides and its interaction with the prion domain of Sup35 have been performed by the Shorter laboratory [67]. These studies have furthered our understanding of the role of Hsp104 in fragmenting Sup35 amyloid [36,68] and have identified different conformations of Hsp104 with bound ATP and ATP analogs [67]. The structure of these different Hsp104 conformations has been determined using cryo-electron microscopy by the Southworth laboratory [69,70]. However, even though these biochemical studies have greatly expanded our knowledge of how these individual proteins work, they still do not clarify the complex protein interactions that occur in the cell during prion curing.

## 7. Concluding Remarks

This paper has focused primarily on the curing of the canonical prion proteins that misfold into a β-sheet amyloid conformation. For the most part, by using multiple complementary techniques, a good understanding has been obtained of the curing of these prions under varying conditions. Interestingly, the same protein can sometimes cure a yeast prion by more than one method, that is by inactivation, dissolution, or asymmetric segregation. For example, inactivation or overexpression of Hsp104 both cure [*PSI*^+^] by inhibiting severing of the prion in the former case and causing dissolution or perhaps asymmetric segregation of the prion in the latter case. Furthermore, we also found that overexpression of a truncated Hsp104 fragment from *S. pombe* cured [*PSI*^+^] by asymmetric segregation.

In the end, the same cellular machinery is used in curing the misfolded form of the prion proteins as it does to clear aggregated misfolded non-amyloid proteins. Nevertheless, there are still many questions regarding the curing of the canonical prion proteins. For example, the curing of [*PSI*^+^] is affected by the cochaperones, Sti1 and Cpr7, but the role of these cochaperones in this curing is still unclear [54,63]. Similarly, the proteasome affects curing of [*PSI*^+^] by Hsp104 overexpression even though there is no evidence of ubiquitination of Sup35, which would be expected if the proteasome is involved. It is not even clear whether overexpression of Hsp104 cures weak and strong [*PSI*^+^] variants by the same mechanism, since the curing of these variants differ in their dependence on members of the Hsp40 family [32]. Since there are perhaps many strong and weak variants, the two different proposed models for the mechanism of [*PSI*^+^] curing by Hsp104 overexpression is due to the use of different [*PSI*^+^] variants in the relevant experiments. Surprisingly, however, even though the Tuite laboratory proposed that the curing of [*PSI*^+^] is due to asymmetric segregation and not dissolution, they only observed a significant difference in seed number between mother and daughter cells when the yeast were ~50%, but not ~30% cured [37], which strongly suggests that the early curing is not due to asymmetric segregation.

Similarly, the relationship between Btn2, Cur1, and Hsp42 in curing [*URE3*] is not understood. Imaging of the fluorescently labeled Ure2 prion domain, as well as full-length Ure2, showed that overexpression of Btn2, Cur1, or Hsp42 cures [*URE3*] and causes aggregation of the [*URE3*] seeds [39,42,60]. The Wickner laboratory found that the curing by either Btn2 or Cur1 are independent of each other, but unlike the curing of Cur1, the curing by Btn2 is dependent on Hsp42, while the curing by Hsp42 requires Cur1 [60]. The complex interplay among these molecules is exacerbated by the fact that Cur1 resides in the nucleus, Hsp42 resides in the cytosol, and Btn2 shuttles between the nucleus and the cytosol [42,71,72,73]. Obviously, more work will be needed to sort out the interactions that occur among these proteins during prion curing.

It is important to point out that the different methods of curing are not mutually exclusive, which compounds the problem of identifying the specific pathways responsible for curing. For example, overexpression of Ydj1 cures [*URE3*] by sequestering Ssa1 [29], but in doing so, it also causes aggregation of the [*URE3*] seeds [39]. Therefore, it is not clear how much the curing of [*URE3*] by Ydj1 overexpression depends on inhibition of severing, since severing by Hsp104 requires Ssa1, and how much it depends on asymmetric segregation because Ssa1 causes aggregation of the [*URE3*] seeds [74].

## 8. Future Directions

An emerging field with many unanswered questions is the mechanism of propagation of non-canonical prions that have two conformations, a regular conformation and a prion transmissible conformation that is not an amyloid. Instead of the glutamine/asparagine (Q/N)-rich amyloid domain present in the canonical prions, these new prion proteins contain an intrinsically disordered domain (IDD). As occurs with the canonical prions, the altered conformation of these IDD-prions is self-templating, which, in turn, enables this altered conformation to be passed from mother to daughter cells. This new class of prion proteins was discovered by the Jarosz laboratory using a screen in which they expressed proteins at high concentrations under stress conditions in yeast [75]. From this screen, 46 proteins were found to maintain an altered conformation under both normal growth conditions and endogenous levels of expression. In many cases, the propagation of the prion conformation is chaperone-dependent, but in contrast to the canonical prions, the propagation of these IDD-prions was not always dependent on Hsp104. Of the 46 IDD-prions, 11 were Hsp104-dependent, 19 were Hsp70-dependent, and two were Hsp90-dependent. The chaperone-dependency of the remaining 14 proteins is not yet known. Since the structures of the prion conformations of these newly identified proteins are not yet understood, it is not clear how they keep propagating.

Proteins with IDD-domains tend to undergo a phase transition from a liquid to a gel under crowding conditions in vitro. Based on this property, one would expect that some of the new prions may undergo such a phase transition in vivo. In fact, [*SMAUG*^+^], a prion recently identified by the Jarosz laboratory [76], forms condensates that self-template in a prion-like manner even though they are not amyloid. [*SMAUG*^+^] is the misfolded conformation of the yeast protein, Vts1, a member of the Smaug family of post-transcriptional regulators that bind RNA. Furthermore, like other proteins with IDD-domains that form condensates in a concentration-dependent manner, condensates formed from purified Vts1 are liquified when the Vts1 concentration is decreased. This suggests that the local concentration of Vts1 in the cell may regulate the curing of [*SMAUG*^+^]. The discovery of the new non-amyloid prions has revolutionized the prion field and undoubtedly will lead to new mechanisms of curing.
